# Impact of moisture on microbial decomposition phenotypes and enzyme dynamics

**DOI:** 10.1093/ismejo/wraf250

**Published:** 2025-11-15

**Authors:** Nicholas J Reichart, Sheryl Bell, Vanessa A Garayburu-Caruso, Natalie Sadler, Sharon Zhao, Kirsten S Hofmockel

**Affiliations:** Earth and Biological Sciences Directorate, Pacific Northwest National Laboratory, Richland, WA 99354, United States; Earth and Biological Sciences Directorate, Pacific Northwest National Laboratory, Richland, WA 99354, United States; Earth and Biological Sciences Directorate, Pacific Northwest National Laboratory, Richland, WA 99354, United States; Earth and Biological Sciences Directorate, Pacific Northwest National Laboratory, Richland, WA 99354, United States; Earth and Biological Sciences Directorate, Pacific Northwest National Laboratory, Richland, WA 99354, United States; Earth and Biological Sciences Directorate, Pacific Northwest National Laboratory, Richland, WA 99354, United States; Department of Agronomy, Iowa State University, Ames, IA 50010, United States

**Keywords:** soil microbiome, activity-based probes, chitin, carbon use efficiency

## Abstract

Soil organic matter decomposition is a complex process reflecting microbial composition and environmental conditions. Moisture can modulate the connectivity and interactions of microbes. Due to heterogeneity, a deeper understanding of the influence of soil moisture on the dynamics of organic matter decomposition and resultant phenotypes remains a challenge. Soils from a long-term field experiment exposed to high and low moisture treatments were incubated in the laboratory to investigate organic matter decomposition using chitin as a model substrate. By combining enzymatic assays, biomass measurements, and microbial enrichment via activity-based probes, we determined the microbial functional response to chitin amendments and field moisture treatments at both the community and cell scales. Chitinolytic activities showed significant responses to the amendment of chitin, independent of differences in field moisture treatments. However, for other measurements of carbon metabolism and cellular functions, soils from high moisture field treatments had greater potential enzyme activity than soils from low moisture field treatments. A cell tagging approach was used to enrich and quantify bacterial taxa that are actively producing chitin-degrading enzymes. By integrating organism, community, and soil core measurements we show that (i) a small subset of taxa compose the majority (>50%) of chitinase production despite broad functional redundancy, (ii) the identity of key chitin degraders varies with moisture level, and (iii) extracellular enzymes that are not cell-associated account for most potential chitinase activity measured in field soil.

## Introduction

Drought and precipitation events can significantly influence both the physical and biological characteristics of soil, leading to unpredictable impacts on soil microbiome function [1–4]. Historical fluctuations in soil moisture directly influence the decomposition rate of soil organic matter (SOM) by altering microbial community composition and the accessibility of carbon resources to microorganisms [5–7]. This accessibility is influenced by microbial composition and their ability to move toward nutrient hotspots [8]. Consequently, nutrient cycling undergoes transformation due to shifts in microbial enzymatic activity [5]. The formation of micro-habitats and biogeochemical hotspots in response to water stress further results in spatially distinct metaphenomes [9]. Despite extensive studies on enzyme activity associated with decomposition at the bulk soil scale [10, 11], the specific phylogenetic details and community interactions involved in substrate access, enzyme production, and nutrient cycling remain largely unsolved.

In complex microbiomes, like those found in soil, microbial communities exhibit functional redundancy, cross-feeding, and competition during decomposition, reflecting access to microbial substrates [12, 13]. This is because SOM degradation requires a suite of enzymes to produce bioavailable carbon and nitrogen. For example, chitin degradation has been used as a model process to understand microbial mediation of SOM decomposition [14, 15] as it provides a significant source of carbon and nitrogen and, therefore, represents an essential cycling hub for both elements [16]. A subset of microbes produces endo-active chitinase that decreases chitin fibril length and yields oligomers. As oligomer concentrations increase (due to substrate concentrations or diffusion), more community members produce exo-active N-acetyl glucosaminidase (NAGase) to yield dimers of chitobiose and monomers of NAG for cellular transport and subsequent use in central metabolic processes. Because of the complexity and importance of SOM decomposition, deciphering the microbial populations that are involved in decomposition and nutrient cycling will advance the understanding of community function across soil ecosystems.

The community enzyme responses to substrate access have important feedback loops that influence SOM decomposition, resource acquisition, and carbon use efficiency (CUE) [17]. Organismal responses to moisture and substrates contribute to taxon-specific growth and respiration rates, leading to community shifts in microbial allocation to biomass. Identifying the generalizable mechanisms of how soil microorganisms coordinate their enzyme production to contribute to heterotrophic decomposition and the reciprocal shifts in CUE can be complex [18, 19]. The influence microbial communities have over SOM cycling brings together the understanding of CUE as the net shift of microbial allocation to biomass (C storage) or CO_2_ (C release) [20]. However, the study of SOM decomposition via a model process can target the underlying phenotypes and metaphenomes that regulate decomposition and CUE [9].

Identifying taxon-specific microbial responses that scale to influence community decomposition and CUE requires methods that bridge from the molecular to the bulk scale. Microbial decomposition of SOM has largely been measured via bulk enzyme assays [21, 22]. Typical soil enzymatic assays query for existing enzymes, intra- and extracellularly, measure bulk potential rates, cannot discriminate between persistent or recently produced enzymes, and lack the ability to link function to taxonomic identification. To address these limitations, we synthesized activity-based probes (ABPs) designed to target active chitinolytic enzymes with an affinity towards chitotriose (Chi3-ABP) or N-acetyl glucosamine (NAG-ABP) [23]. Activity-based protein profiling (ABPP) is a technique that uses small molecule chemical probes to target enzymes of a given function in complex proteomic systems. Several approaches for downstream analysis of labeled targets include fluorescence-activated cell sorting (FACS), microscopy, or SDS-PAGE, whereas other affinity-based enrichment strategies allow for enzyme pull down followed by identification by LC–MS. Recent advances in applying ABPP coupled to FACS have been demonstrated using samples extracted from complex microbial communities in the murine gut [24, 25], a hot spring [26], and soil [27]. Here we present the first application of ABP-FACS in tandem with biogeochemical assays, providing a direct linkage of enzyme activity to microbial taxonomic identity, enabling the connection of phenotype to community function.

Here, we examined soil moisture effects on both community and organismal scale chitin decomposition and community function. We hypothesize that field moisture regimes govern decomposition rates in soil and is a stronger factor of microbial function than substrate abundance. We predict greater levels of enzyme activity, chitin decomposition, and microbial growth in wet sites due to increased spatial connectivity and substrate access [28]. Using a lab incubation study, we analyzed how environmental conditions of field moisture and substrate concentration influence accessibility, as measured by respiration rates, enzyme function, and microbial community composition. Additionally, we hypothesize that only a few microorganisms produce endochitinases, and many microorganisms produce NAGases and benefit from the initial complex breakdown of chitin [29]. We aim to identify key community members producing enzymes involved in chitin decomposition. By combining soil incubation measurements of potential enzyme activity and respiration concurrent with ABP enrichment, we identify how chitin-decomposing populations respond to conditions of substrate concentration and soil moisture. Our analyses reveal distinct microbial populations and enzymatic function dependent on the carbon and moisture treatments, highlighting the plasticity of the soil microbiome to changing conditions.

## Materials and Methods

Additional details are provided in the supplemental online materials.

### Field site description and incubation

The field site is operated by the Washington State University-Irrigated Agriculture Research and Extension Center, located in Prosser, WA (46°15’04”N and 119°43’43"W). The soil from the field was characterized as marginal Warden silt loam with low organic matter [30]. In 2018 the trial was established with two varieties of perennial tall wheatgrass (*Thinopyrum ponticum*); including “Alkar,” a northern ecotype bred for the Pacific Northwest was sampled for this study. In 2019 field moisture treatments were imposed at four levels (100%, 75%, 50%, and 25% field water capacity) to create plots with differing drought stress. On October 18^th^, 2022 just after the fall harvest, and after four years of differing field moisture treatments, soil was collected from plots planted with the Alkar cultivar of Tall Wheatgrass. Samples were collected from two long-term irrigation plots for low moisture (plot 23, 25% field capacity) and high moisture (plot 42, 100% field capacity) treatments to examine the effects of chitin amendment on historical moisture regimes. Five replicate cores (5 cm diameter) from 0–15 cm depth were collected from each plot to achieve sufficient mass for the incubation scheme without increasing sampling depth. Irrigation water used for the field site was collected from the adjacent irrigation channel, which is fed from the Yakima River, and used for moisture control in the lab-based incubations. An average soil temperature of 15.3°C for the week preceding sample collection was calculated from soil probes at 18 cm depth in 12 replicate locations at the field site.

Replicate cores were combined and gently homogenized to maintain aggregation. Roots, plant detritus, and rocks were picked out with sterile tools. Gravimetric water content was collected by drying 10 g soil at 60°C for 48 h. For each moisture level (25% and 100%), 80 g of field moist soil was weighed into 10 autoclaved sterile replicate 237 ml wide mouth canning jars (20 jars total). All jars were preincubated at 15.3°C for 4 days. The experiment was initiated by adding 1000 ppm chitin (Bean Town Chemical, Hudson, New Hampshire) (1 mg/g dry soil) to five replicate jars of each moisture level (25% and 100%) and stirred to homogenize with sterile spatulas. Ten replicate non-chitin control jars (five at each moisture level) were also stirred for consistency in disturbance across treatments. All samples (*n* = 20, Supplemental Table 1 for a description of all sample conditions) were brought back to field moisture with 0.22 μm sterilized irrigation water from the site. Samples were incubated at 15.3°C for 7 days. Subsamples for biomass measurements and activity-based probing were placed in appropriate containers and stored at 4°C. Additional subsamples for chitin measurements and extracellular enzyme activity were stored at −20°C. Finally, subsamples for DNA extraction and for archival samples were collected and stored at −80°C.

### Soil respiration and microbial biomass

Jars were capped with canning jar lids modified with butyl septa. Measurements for CO_2_ were performed daily by removing 1 ml headspace in a precision gas syringe and injected manually via a valved sample injection loop system into a Licor LI7000 (Licor, Lincoln, Nebraska). After each daily sampling, jars were uncapped for 5 min inside a biological safety cabinet to equilibrate to room air and then recapped for the following day’s measurement. Cumulative respiration among treatments were calculated as the sum of the daily CO_2_ produced (μg CO_2_-C per g_dry_wt_soil) over the 7-day incubation. For salt extractable and microbial biomass C and N pool quantification, we used sequential chloroform fumigation extraction (CFE) [31]. Samples were frozen at −20°C for storage until total organic carbon (TOC), total nitrogen (TN) analysis.

### Soil chitin extraction and quantification by high-performance liquid chromatography

Pre- and post-incubation chitin concentrations in the soil were aextracted and quantified as previously described [32, 33] . Briefly, free amino sugars were removed prior to acid assisted chitin hydrolysis to glucosamine using 100 mg of freeze-dried soil for each sample. A homocysteic acid internal standard (5 μm) was added to the samples and prepared D-(+)-glucosamine HCl standards (0–12.5 μM). The samples and standards were derivatized with FMOC-Cl in acetone and cleared of any particulates by passing each sample through a Thomson 0.2 μm filter vial. dDerivatized glucosamine peaks were detected using a Shimadzu HPLC 20A with a CTO-20A baseline detector, and SIL-20A HT autoinjector. An average sum of the external calibration factor was calculated using the standards concentrations to solve for the samples’ glucosamine concentrations.

### Bulk soil enzyme potential assays

A total of eight potential extracellular enzyme activities were measured from soil samples [34]. These included hydrolytic enzymes for carbon, nitrogen, and phosphorus acquisition (Supplemental Table 5 for substrates, concentrations, functions, and EC). Saturating substrate concentrations were determined during initial testing of Km and linearity of the reactions. Activities were calculated following previously published protocols [21, 22], except that net fluorescence, quenching, and emission coefficients were calculated at 1 and 3 h, and activity was then calculated by the change over time between the two measurements. This assay measures potential rates. Potential enzyme assays are run under saturating substrate concentrations, and in a soil slurry which maximizes physical dispersion of both enzymes and substrates and therefore do not reflect the rate of the reaction occurring in a soil, but rather the size of the enzyme pool.

### Biogeochemical statistical analyses

All statistical analyses for the biogeochemical measurements [35] were performed in R (version 4.4.1) with a significance statement of *P* < .05. The relationship between the cumulative respiration and incubation time was evaluated using ordinary least squares regressions (function “lm”). Normal distribution of the data was confirmed using the Shapiro–Wilk test. Non-normally distributed data (bulk soil chitin activity and microbial biomass carbon) were transformed by Box-Cox transformation (function “BoxCoxTrans” in the caret package) and rechecked to confirm normality prior to analysis. Comparison across treatments was assessed using ANOVA analysis for moisture, chitin addition, and the interactions. Tukey HSD *post hoc* tests were performed on significant comparisons.

### Nycodenz extraction of microbial cells from soil

Microbial cells were collected from the soil using a Nycodenz (Serumwerks Bernburg AG, Bernburg, Germany) gradient with a slight variation of sample volume [27]. A subsample for 16S rRNA gene sequencing was collected from the Nycodenz extracted microbes for each sample to compare the microbial composition to the original community in the soil. We acknowledge that the Nycodenz extraction method may inflate or exclude the presence of certain taxa over others. For example, previous studies have shown underrepresentation of *Actinobacteriota* and *Firmicutes* and that cell size, weight, and morphology all have impact on the recoverability of cells through density gradient centrifugation [36, 37]. To address this intrinsic bias, throughout the processing, subsamples were collected for 16S rRNA gene sequencing to track any methodological biases. Both the subsamples from the Nycodenz extraction as well as the original, bulk soil samples had DNA extracted with the Zymo Quick Fecal/Soil DNA Miniprep kit (Zymo Research, Irvine, California, cat no. D6010) according to manufacturer's instructions. The Nycodenz extracted samples were distinct from bulk DNA extracted from soil regarding community composition and exhibited a decrease in observed taxa richness as well as Shannon Index (Supplemental Fig. 1).

### Extracted microbial kinetic assay

Kinetic assays were set up using 100 μL (10% of the volume) of the Nycodenz extracted microbial populations (as described above). Cells were incubated with 4-Methylumbelliferyl β-D-N,N’,N″-triacetylchitotriose (Chitin, Supplemental Table 5) or 4-Methylumbelliferyl N-acetyl-β-D-glucosaminide (NAG, Supplemental Table 5) and were continuously measured for fluorescence on a BioTek H1 Synergy plate reader (Agilent, Santa Clara, California) measuring once per hour for 40 h.

### Application of activity-based probes for enrichment through fluorescence-activated cell sorting

To assay microbial enzyme production at the cellular scale, the remaining volume from the Nycodenz extracted cells was used for ABP labeling. All samples were extracted the day prior to cell sorting and were stored at 4°C in between extraction and sorting. ABPs were developed to target chitotriose (Chi3-ABP) or N-acetyl glucosamine (NAG-ABP) [23]. Each sample was split into five fractions: Chi3-ABP, NAG-ABP, Syto9 (Invitrogen) nucleic acid stain control, Syto63 (Invitrogen) nucleic acid stain control, and a no-fluorescence control. Two Syto stains were used due to the different fluorophores conjugated to the ABPs. The corresponding ABP was added to a final concentration of 100 μM from 10 mM stocks. Probe labeling occurred in the dark, incubating at 25°C shaking at 500 RPM for 2 h. Each sample was suspended in a final volume 3X the starting volume and filtered through a 35 μm cap into a flow cytometry tube. For Syto9 and Syto63 controls, a final concentration of 0.5 μM of Syto dye was used.

Enzyme-producing microbial populations labeled with fluorescent probes were separated from non-enzyme producing populations via FACS. A Sony SH800 (Sony Biotechnologies) with four lasers configuration was used with forward scatter as a threshold. Detectors were optimized for intensity on the first day of sorting and maintained throughout the experiment. Sorting gates were determined in a nested fashion, starting with forward scatter area by back scatter area (FSC-A × BSC-A), followed by forward scatter height by forward scatter width (FSC-H × FSC-W) to select for appropriately sized cell events (Supplemental Fig. 2).

At the beginning, middle, and end of each day, sheath fluid was collected from the sheath stream to monitor for sample line contamination. For each ABP-labeled sample, four replicates of 50 000 ABP positive events were sorted into 1.5 ml tubes using the normal sorting mode. At the completion of each sorting day, all samples were processed for cell lysis using Invitrogen Direct to PCR Lysis Buffer (Cat No. A44647500). The samples were lysed following manufacturer’s recommendations. Samples were stored at −20°C, until processing for PCR of the 16S rRNA gene.

### Amplicon PCR and Illumina sequencing

The v4 region of the 16S rRNA gene was amplified using the 515f [38] and 806r [39] primer set following the Earth Microbiome Project (EMP) protocol [40] as a one-step PCR with unique barcodes on the forward primer and Illumina adapters. PCR was conducted with a final volume of 50 μL. All PCR products were quantified in triplicate using Quant-iT Picogreen dsDNA assay (Invitrogen). Samples were pooled at an equimolar volume of 200 ng per sample. Standard Illumina library preparation was followed and a 15% PhiX spike-in was used to load the v2 500 cycle paired end MiSeq System (Illumina) reagent cartridge.

### Amplicon data analysis

Demultiplexed amplicon reads [41] were exported from the MiSeq System and processed using QIIME2 (version 2021.4, [42]). The reverse reads were low quality, so all subsequent analyses were performed with the forward reads. The imported reads were denoised through sequencing, trimming, and quality filtering before assigning amplicon sequence variants (ASVs) using the DADA2 [43] plugin within QIIME2. The forward reads were trimmed 13 bases on the 5′ end and truncated at base 181 to maintain read quality. Taxonomy was assigned in QIIME2 using the SILVA database (version 138) trained to the v4 region of the 16S rRNA gene.

Taxonomy assigned reads were processed using R (version 4.4.1, [44]). Decontam [45] was applied using the Prevalence model and threshold 0.6 to remove potential contaminant ASV reads from the dataset by comparing the occurrence of ASVs in true samples or control samples. PCR negatives, blank FACS sheath fluid collections, and DNA kit extraction blanks were used from each day of processing as control samples. Sequencing reads were rarefied to 15 443 reads per sample to account for unevenness of sequencing depth across samples prior to further analysis. Phyloseq [46] was used to calculate the alpha-diversity of the samples using measurements for observed taxa for richness and Shannon Index for evenness among samples and between incubation time points. The community composition of the samples was analyzed via relative abundances plotted using nonmetric multidimensional scaling (NMDS) of Bray–Curtis dissimilarity. A PERMANOVA test was used to determine significance among treatment groups using Bray–Curtis dissimilarity, perm = 999, and Holm as a *P*-value adjustment. DESeq2 [47] was used to determine the enrichment of microbes via ABP-FACS between the chitin amended samples for each moisture group. A minimum log fold change of 1 and false discover rate of 0.1 were used to determine enriched taxa, considering a critical read base mean to minimize variations due to sequencing errors.

## Results

### Respiration of microbial incubations was impacted by chitin amendment

**Figure 1:**
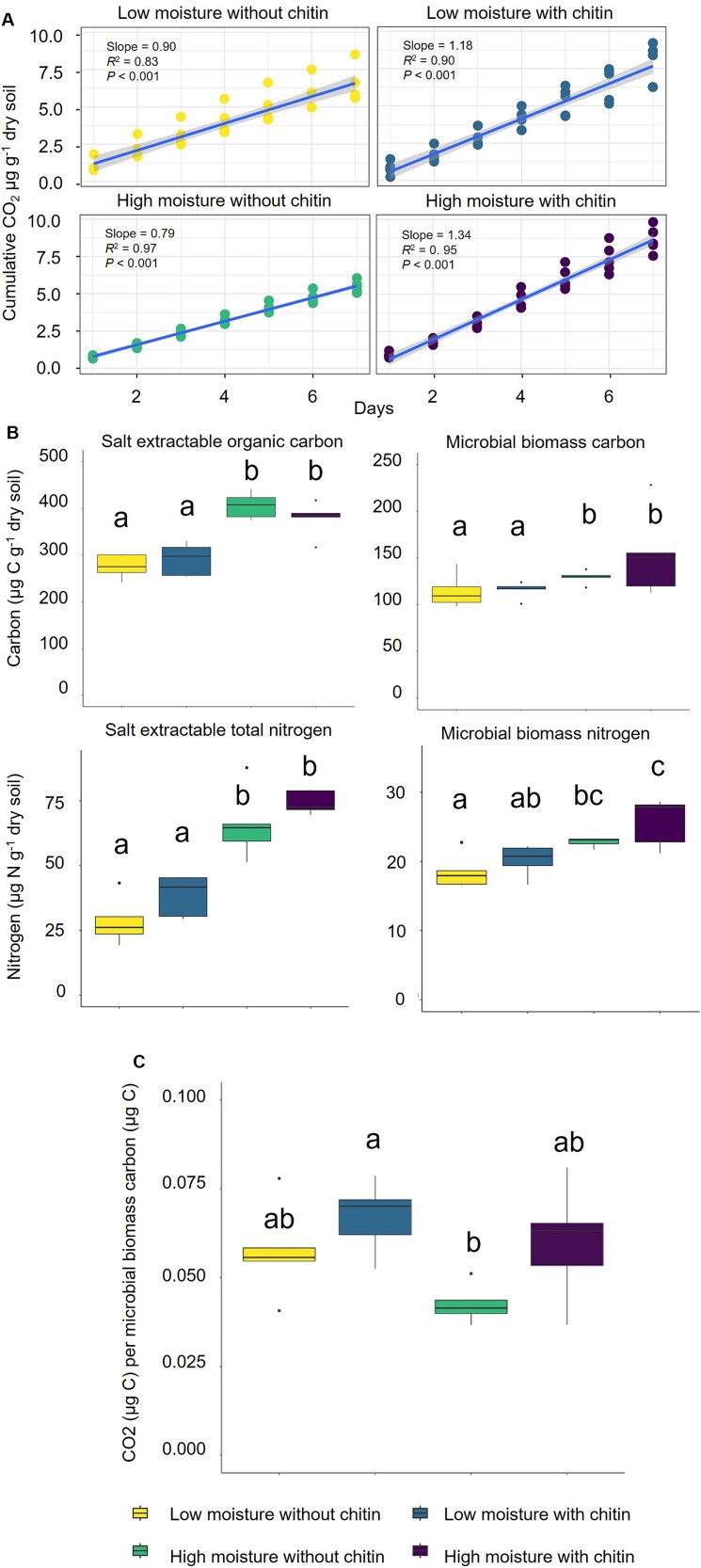
Soil biogeochemistry measurements reveal the impact of moisture and chitin amendment. (A) Cumulative soil respiration over the seven-day incubation with mean and standard deviation plotted for biological replicates (*n =* 5). (B) Salt extractable and microbial biomass carbon and nitrogen for each incubation condition. (C) Carbon use efficiency by proxy of the ratio between the respired carbon in CO_2_ related to the microbial biomass carbon. Different lowercase letters indicate significance according to ANOVA and Tukey honest significant differences *post hoc* testing (*P* < .05).

Prior to the start of the incubation, gravimetric water content (GWC) of field fresh soil was 15.6% for the low moisture plot and 18.3% for the high moisture plot, corresponding to 42% and 51% water holding capacity, respectively. The high moisture with chitin amendment treatment had the highest production of CO_2_ over the seven-day incubation period at a rate of 1.34 μg CO_2_ g^−1^ dry weight of soil day ^−1^ (Fig. 1). All treatment types had a significant cumulative increase in respiration over the course of the incubation (*P* < .001). Chitin amendment had a significant impact (*P* = .002) on microbial cumulative respiration of CO_2_, whereas moisture level did not (*P* = 0.914, Fig. 1A, Supplemental Table 2). This is indicative that the response seen in chitin amended samples is due to the presence of additional chitin in the system causing excess carbon relative to the demand for biomass. Additionally, the accumulation of CO_2_ over time was linear in all four treatment conditions, suggesting no resource limitation during the seven-day incubation. Previous microbial incubation studies reported peak CO_2_ emission between 10 and 14 days of incubation followed by a sharp decline in production, suggesting our incubation time length was appropriate to capture the initial microbial response for chitin decomposition [15]. Chitin amendment did not impact salt-extractable or microbial biomass carbon but significantly increased salt-extractable nitrogen (*P* = .04) and microbial biomass nitrogen (*P* = .05). Samples with high moisture contained significantly more bioavailable and biomass carbon and nitrogen independent of chitin amendment (Fig. 1B, ANOVA values provided in Supplemental Table 3).

To evaluate the chitin decomposed over the incubation, chitin was extracted, hydrolyzed, and measured as glucosamine concentration. Within treatment groups, glucosamine concentrations were not significantly different between Day 0 and Day 7 (Supplemental Fig. 3, Supplemental Table 4). When analyzing the differences in glucosamine concentration on Day 0, chitin amendment and moisture level had a significant impact (*P* < .001 and *P* = .018, respectively). The significant impact of chitin amendment and moisture level was also present at the end of the incubation on Day 7 (*P* = .006 and *P* = .004, respectively) (ANOVA values provided in Supplemental Table 4).

### Differential effects of moisture on enzyme activity

**Figure 2:**
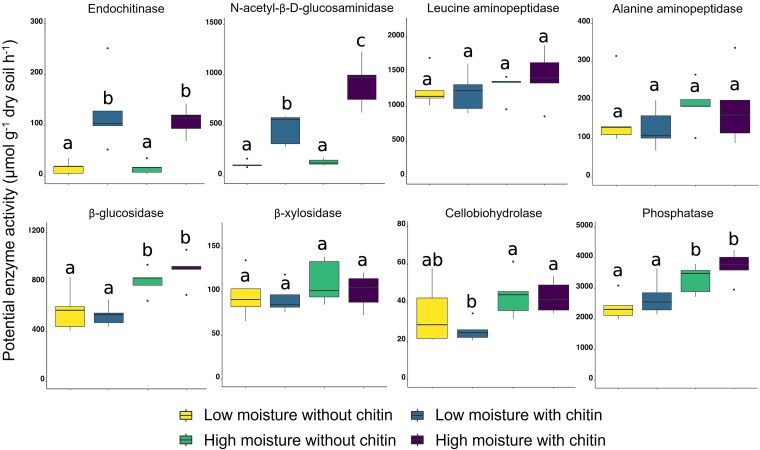
Potential soil enzyme activities as impacted by moisture and chitin amendment after seven days. Lowercase letters indicate significance according to ANOVA and Tukey honest significant differences *post hoc* testing (*P* < .05). The scale varies among plots.

Potential enzyme activity was measured in the bulk soil following the seven-day incubation as a proxy of resource demands in response to moisture and substrate addition (Supplemental Table 5). Mean phosphatase activity across all treatments exceeded the next most active enzyme (leucine aminopeptidase) by more than 2-fold and the lowest activity enzyme (cellobiohydrolase) by two orders of magnitude. AP activity was followed by leucine aminopeptidase, β-glucosidase, N-acetyl-β-D-glucosaminidase, alanine aminopeptidase, β-xylosidase in decreasing order, and lastly endochitinase, and cellobiohydrolase which target the interior bonds on large polysaccharides. Chitin addition significantly increased the enzyme activity for N-acetyl-β-D-glucosaminidase (7-fold increase, *P* < .001,) and endochitinase (5-fold increase, *P* < .001) relative to samples without chitin amendment. Of these two enzyme assays, only N-acetyl-β-D-glucosaminidase was impacted by soil moisture (*P* = .002) which had 5-fold higher activity than endochitinase in chitin amended soils. As a main effect, greater soil moisture resulted in greater activity than the low moisture samples for β-glucosidase (*P* < .001), cellobiohydrolase (*P* = .013), and phosphatase (*P* < .001), with chitin amendment having no effect on these activities (Fig. 2, Supplemental Table 6). No treatment effects were detected for potential β-xylosidase, leucine aminopeptidase, or alanine aminopeptidase activity.

**Figure 3:**
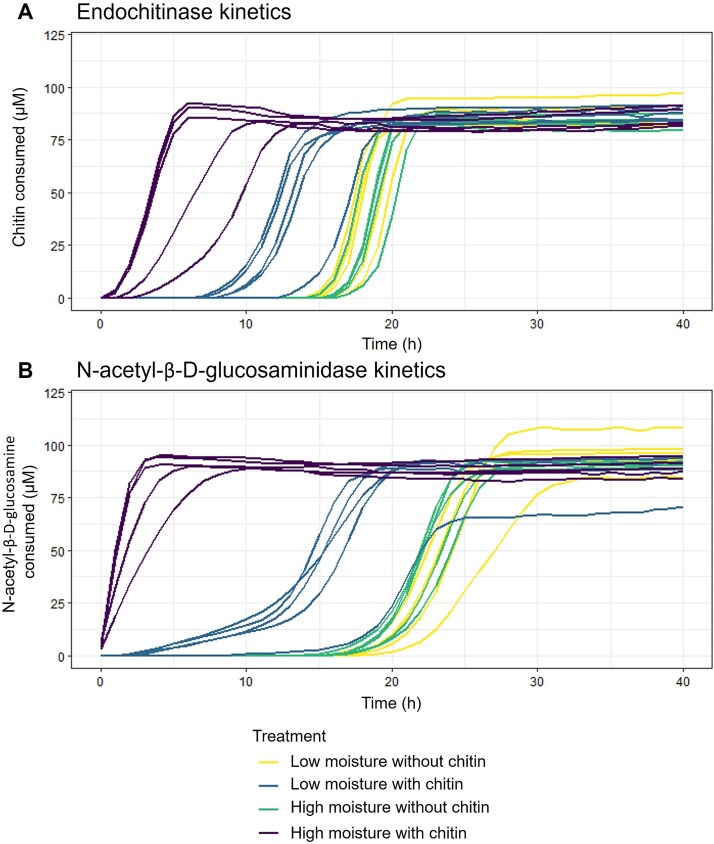
Kinetic curves of methylumbelliferyl linked substrate incubated samples. Each line is the average of three kinetic replicates per sample. (A) 4-Methylumbelliferyl β-D-N,N′,N′′-triacetylchitotriose (endochitinase) samples. (B) 4-Methylumbelliferyl N-acetyl-β-D-glucosaminide samples.

To quantify the enzymatic response of intracellular or cell bound enzymes that would be detected by our ABPs in later processing, we applied the potential soil enzymatic assays as used above for endochitinase (Chitotriose-MUB) and N-acetyl-β-D-glucosaminidase (NAG-MUB) on the Nycodenz extracted cellular fraction. By using cells extracted from the soil matrix, we were able to run the activity assays as kinetic assays, measuring cumulative enzymatic activity every hour over 40 h (Fig. 3). Additionally, because the samples were subjected to centrifugation washes during extraction with Nycodenz, these enzymatic assays were a direct result of intracellular or newly secreted enzymes and not persistent or relic enzymes in the soil potentially detected by potential enzyme activities methods. To test for treatment effects on the rate of enzyme production and activity, we compared how long it took for half of the chitotriose- or NAG-MUB substrates to be depolymerized. On average, soil microbes extracted from the high moisture with chitin amendment incubations were the fastest to depolymerize half of the provided Chitotriose-MUB substrate (50 μM) at a time of 5 h. This was followed by low moisture with chitin after 14 h and both moisture treatments without chitin after 19 h of incubation. This general trend of a rapid response by chitin amended samples was followed for samples incubated with NAG-MUB. High moisture with chitin was first to cross the 50 μM threshold after only 1 h of incubation, followed by low moisture with chitin after 17 h and high moisture without chitin and low moisture without chitin after 23 and 24 h, respectively (Fig. 3).

To reconcile the differences of these assays, we compared the rate of substrate conversion in the bulk enzyme assays to the cell-extracted kinetic enzyme assays for endochitinase and N-acetyl-β-D-glucosaminidase (Table 1). The bulk enzyme assays had higher substrate turnover than the kinetic assays. For endochitinase measurements, the bulk assay rates were 4.95-fold higher (SE ± 1.34) than kinetic assays. The discrepancy between bulk and kinetic assays was higher for N-acetyl-β-D-glucosaminidase activity with 24.69-fold higher rates (SE ± 4.12) in bulk versus kinetic assays. Sample rates were more variable in the bulk enzyme assays compared to the kinetic assays (Table 1). Bulk enzyme assays had higher rates for N-acetyl-β-D-glucosaminidase compared to endochitinases, whereas the kinetic assays had a similar magnitude of rates for both enzyme functions. The kinetic assays had a similar magnitude of rates for both enzyme classes. The kinetic assay showed substrate turnover earlier in the incubation for N-acetyl-β-D-glucosaminidase compared to endochitinase (Fig. 3).

**Table 1:** Comparison of enzymatic activity between bulk kinetic assay for total soil and kinetic assay on Nycodenz extracted cells.

	Bulk potential enzyme assay activity (mean ± SE)(μM g-1 h-1)		Kinetic enzyme assay activity (mean ± SE)(μM g-1 h-1)	
	Chitinase activity	NAGase activity	Chitinase activity	NAGase activity
Low moisture without chitin	16.24 (6.00)	116.18 (14.43)	8.79 (0.21)	6.75 (0.22)
Low moisture with chitin	126.90 (33.51)	466.68 (66.76)	11.74 (0.65)	9.80 (0.67)
High moisture without chitin	116.18 (14.43)	139.08 (14.14)	8.61 (0.22)	7.14 (0.15)
High moisture with chitin	107.90 (12.45)	918.62 (104.20)	33.10 (5.37)	91.67 (20.41)

### Moisture and chitin amendment impact soil bacterial diversity

**Figure 4:**
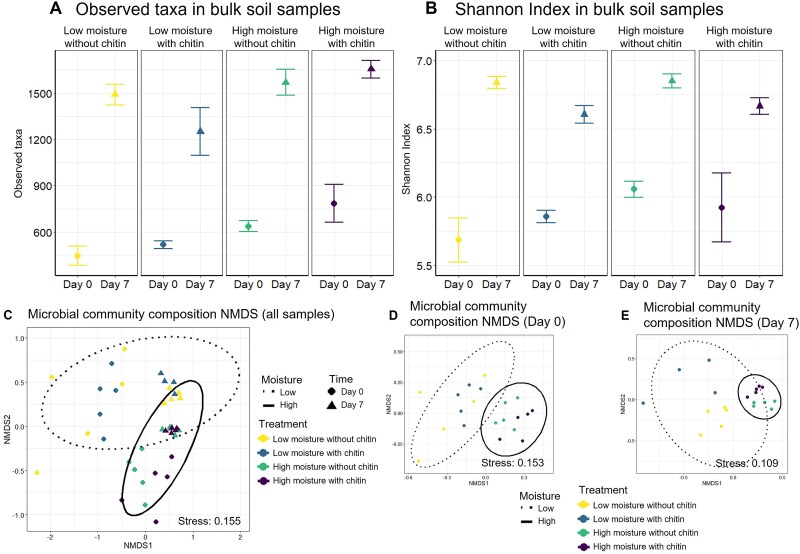
Changes in microbial diversity and composition from bulk soils across moisture and chitin treatments. Diversity measurements of 16S rRNA gene sequencing for bulk soil incubations compared from Day 0 to Day 7. (A) Averaged observed taxon count for each incubation treatment; error bars represent standard error. (B) Averaged Shannon index for each incubation treatment; error bars represent standard error. (C) Nonmetric multidimensional scaling of Bray–Curtis dissimilarity of community composition for relative abundance of the total incubated samples. (D) Sample composition at the beginning of the incubation. (E) Sample composition at the conclusion of the seven-day incubation. Ellipses represent 95% confidence intervals.

We explored the impacts of moisture and chitin amendment on soil bacterial diversity using 16S rRNA gene sequencing. We sequenced 235 samples in total, including the bulk soil, Nycodenz fractions, probe sorted samples, as well as controls. This resulted in 11 320 554 reads at an average depth of 48 172 reads per sample. In total 34 568 ASVs were detected. The most abundant phlya in the bulk soil at Day 0 samples were *Proteobacteria* (30.5%), *Acidobacteriota* (14.0%), *Actinobacteriota* (12.8%), *Bacteroidota* (10.2%), and *Planctomycetota* (4.1%). The most abundant phyla at Day 7 were *Proteobacteria* (30.8%), *Actinobacteriota* (19.0%), *Acidobacteriota* (12.6%), *Firmicutes* (8.3%), and *Bacteroidota* (5.6%). Across the seven-day incubation, we observed significant increases within each treatment for α-diversity in both the richness of observed taxa and Shannon Index measurements (Fig. 4A and B, respectively, *P* < .05, Supplemental Table 7). On Day 7, chitin amended samples had greater alpha diversity of observed taxa richness in both low and high moisture samples compared to the unamended samples (*P* = .05). Chitin amendment also impacted the Shannon Index for low and high moisture treatments (*P* = .05). Additionally, the community composition of the treatments shifted across the incubation from Day 0 to Day 7 with *Chitinibacter* increasing in incubations with chitin (*P* < .05), a consistent decrease of *Phyllobacterium* in all treatments (*P* < .05), and a nonsignificant increase of *Cellvibrio* in chitin amended samples (*P* > .05, Supplemental Fig. 4).

When analyzing the beta-diversity for all samples from the soil incubations including both time points (Fig. 4C, Supplemental Table 8), the variables for time (*R*^2^ = 0.14, *P* = .001) and moisture (*R*^2^ = 0.07, *P* = .001) caused significant changes to the community composition, whereas the chitin amendment did not (*R*^2^ = 0.03, *P* = .08). We separated the two time points and reanalyzed the relationship of community composition within each time point. For Day 0 samples, only moisture influenced the community composition (*R*^2^ = 0.09, *P* = .001), reflecting the distinct initial communities in the field experiment between the two irrigation treatments (Fig. 4D). For Day 7 samples, both moisture (*R*^2^ = 0.21, *P* = .001) and chitin (*R*^2^ = 0.09, *P* = .01) influenced the composition with more compositional variation being explained by the moisture treatment. Together these results demonstrate that the compositional differences in response to field moisture treatments, seen in Day 0 samples, were maintained in the incubation, and a microbial bloom in response to chitin amendment was detected after the seven-day incubation (Fig. 4E).

### Microbial populations from activity-based probing

**Figure 5:**
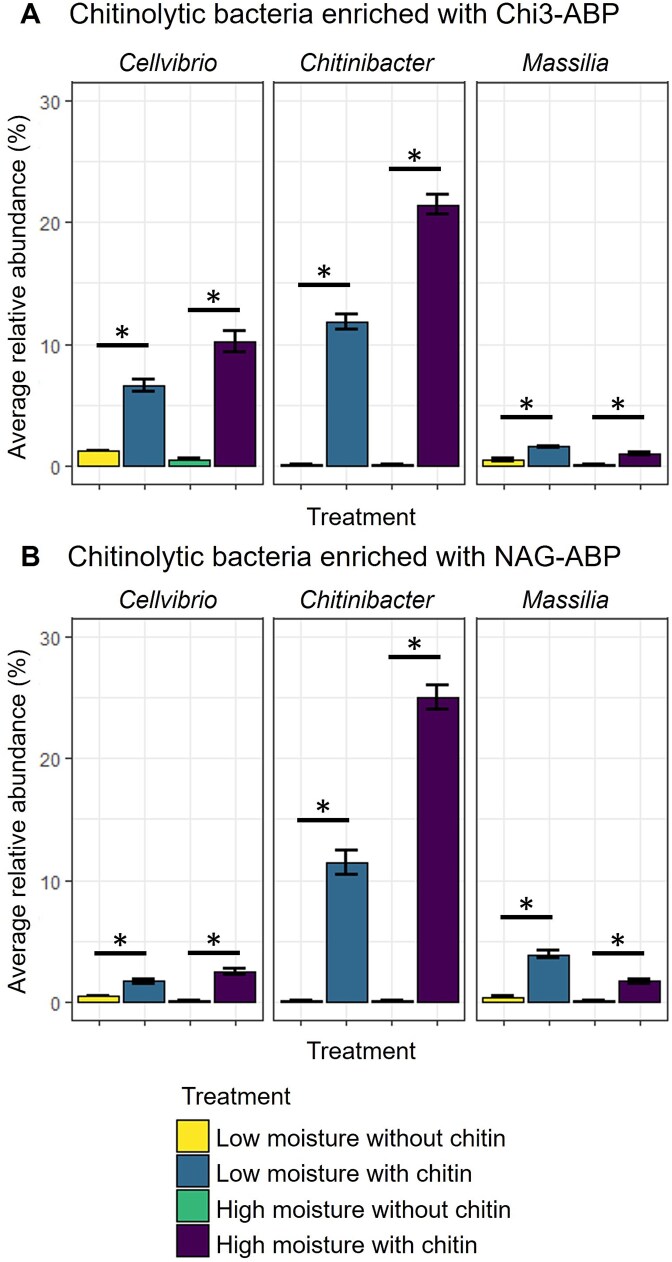
Varied abundance of chitinolytic genera in response to chitin treatment as determined by activity-based probe labeling. (A) Mean (± SE) relative abundance of taxa enriched using Chi3-ABP between moisture and chitin treatments. (B) Mean (± SE) relative abundance of taxa enriched using NAG-ABP between moisture treatments. Significance from DESeq2 analysis denoted by “^*^” (*P* < .001, LFC > 2) only comparing chitin amendment within a moisture treatment. Error bars represent standard error.

We applied two different ABPs to target active microbes within the soil microbiome expressing enzymes acting upon either chitotriose (Chi3-ABP) or N-acetyl glucosamine (NAG-ABP) and sorted these microbial populations with fluorescent-labeling via FACS. The ABPs were able to bind to enzymes across diverse taxonomies in samples with and without chitin amendment. Samples amended with chitin in both moisture conditions had lower richness and diversity evenness compared to the unamended samples. At the family level, 51 unique taxa were detected with over 1% relative abundance across all samples with the NAG-ABP. Conversely, only 44 unique taxa were detected at the same threshold in the Chi3-ABP samples (Supplemental Fig. 5). These results suggest there is plasticity in the metabolism of a subset of the microbial populations in the soil that respond to inputs of chitin regardless of soil moisture. The microbes that were labeled with each probe were compared at the genus taxonomic level to identify specific taxa enriched in chitin amended incubations in either moisture level. A multitude of taxa were labeled with ABPs, however, three taxa, *Cellvibrio*, *Chitinibacter*, and *Massilia*, were identified using log fold change analysis to be increased in chitin amended samples compared to without chitin incubations (adjusted *P* < .01). *Cellvibrio* and *Chitinibacter* populations were more abundant via ABP-FACS for high moisture samples, whereas *Massilia* was more abundant in the low moisture samples further supports variability of soil microbes responding to soil moisture (Fig. 5). Taxa in the *Pseudomonadaceae* family were highly abundant regardless of the incubation condition (Supplemental Fig. 5). However, there were no significant changes in abundance when comparing moisture treatments for *Pseudomonadaceae* members. A small subset of taxa at the genus level composed the majority of the sequencing reads for the ABP-FACS populations. For the Chi3-ABP samples averaged across all conditions, the seven most abundant taxa made up the top 51.9% of reads (*Pseudomonas, Chitinibacter, uncultured Vicinamibacterales, Vicinamibacteraceae, Cellvibrio,* and *Emticicia*). For the NAG-ABP samples, eight taxa made up the top 51.8% of reads (*Pseudomonas, Chitinibacter, A4b, uncultured Vicinamibacterales, Vicinamibacteraceae, uncultured Gemmataceae, uncultured Comamonadaceae,* and *Ellin6067*) (Supplemental Fig. 6).

## Discussion

Evaluating soil community function and nutrient cycling in response to perturbation at multiple scales is critical for gaining a holistic understanding of soil microbial phenotypes. Current understanding of decomposition by soil microorganisms is largely derived from bulk enzyme assays. Although this approach serves to quantify the potential activity of specific enzyme classes in soil samples, it fails to identify specific enzymes or assign functional roles to community members. Here, we use an innovative approach, ABP-FACS, to survey taxon-specific responses to chitin amendment and field moisture treatments and pair these measurements with a panel of bulk enzyme and biogeochemical assays to identify the microbial mechanisms driving bulk soil decomposition. Using ABP-FACS, we demonstrate that although there is widespread chitinolytic functional redundancy among taxa within a soil microbiome, only a few taxa are in an active chitinolytic state at high abundances and may be responsible for driving chitin decomposition at the time of sampling (Supplemental Fig. 6).

### Shifts in microbial functions towards soil carbon cycling in response to moisture and chitin amendments

The effects of substrate access have direct consequences for microbial activity, especially in decomposition and nutrient cycling. The ability of microorganisms to efficiently convert available substrates into biomass is important for both microbiome function [48] and SOM cycling [49]. When microbial depolymerization of substrates is considered in aggregate, at the bulk soil scale, chitin addition stimulates a significant increase of N-acetyl-β-D-glucosaminidase and endochitinase activity. Our study revealed that overall nutrient limitation in our marginal soils results in high rates of enzyme production aimed at nitrogen and phosphorus acquisition (phosphatase and peptidase activity). Because of the inherently low nutrient status, increased soil moisture had no detectable effect on potential enzyme production apart from ꞵ-glucosidase. Instead, high moisture stimulated decomposition and assimilation of carbon and nitrogen more than chitin amendments, with no effect on CUE. The chitin amendment provided the microbial populations with key nutrients, leading to a bloom of activity and growth that was less strongly affected by soil moisture levels. The stoichiometry of the microbial biomass pool also supports nitrogen limitation, with microbial biomass carbon unaltered by chitin amendment, whereas microbial biomass nitrogen increased with chitin amendment. Coupled with the high biomass specific respiration under chitin amendment, our results suggest that the added chitin was used for microbial biomass N and the excess C was respired. Although our methods cannot distinguish residual chitin in the soil from newly formed structural chitin within fungal cell walls, the lack of significant change in chitin recovery throughout the incubation for each treatment, in combination with increased enzyme activity, suggests decomposition products were rapidly incorporated into de novo fungal growth. These findings demonstrate the importance of environmental parameters in driving microbial enzyme production, where the decomposition of substrates reflects stoichiometric demands and the availability of multiple resources to support microbial growth and activity. Bulk enzyme and biogeochemical assays point to complex environmental and nutritional requirements driving community decomposition dynamics that need to be characterized using molecular approaches.

Defining community functions in soil at the time of sampling is challenging in part because of the temporal discrepancy between enzyme production and widely variable extracellular enzyme decay rates [50]. We used bulk enzyme and extracted cell kinetic enzyme assays to provide insights into the treatment effects on chitinolytic enzyme activity of the total soil system and the cellular community members, respectively. Bulk enzyme activity assays measure the combined hydrolytic potential of cell associated, freely diffuse, and particle bound extracellular enzymes in soil samples. Our assays show an excess of enzyme activity in bulk assays compared to kinetic assays which was induced by the addition of chitin, particularly with NAGase. Freely diffuse enzymes tend to lose activity rapidly (<1 day), but particle bound enzymes have been found to maintain stable activity for weeks or longer [51, 52] and absorption to mineral surfaces can occur rapidly [53]. Even in these silt loams, with low reactive minerals, bulk soil assays reveal a significant contribution of relic, or persistent enzymes. These bulk measurements are critical to understanding the total enzymatic potential of the system and are frequently used in ecosystem models [54]. However, these results cannot be directly tied to the metaphenome of the microbial community at the time of sampling. The extracted cell kinetic enzyme assays provide a mechanistic understanding of taxon specific interactions during decomposition. Kinetic assays exclude the freely diffuse and particle bound enzymes and instead tracks community substrate turnover. The kinetic assays showed that cells from the chitin treated communities were primed to produce chitinolytic enzymes earlier in the incubation than the cells from soils that were not treated with chitin (Fig. 3) and the faster response of N-acetyl-β-D-glucosaminidases could indicate that there were more community members with cell associated N-acetyl-β-D-glucosaminidase than with endochitinase or a faster substrate turnover rate with N-acetyl-β-D-glucosaminidase. The combined results comparing bulk enzyme activity to kinetic enzyme activity of Nycodenz extracted cells indicate that by Day 7, the community relied heavily on extracellular enzymes that accumulated over time. At this stage of the incubation, N-acetyl-β-D-glucosaminidases were a bottleneck in chitin processing and required higher activity levels. Determining the response and production of enzymes can lead to better modeling of carbon cycling and dynamics of diffusion for both enzymes and nutrient hot spots in the soil matrix.

Enzyme costs have recently been considered for CUE calculations for models [54] due to their fundamental role in decomposition, substrate assimilation, and subsequent allocation to biomass or respiration. With an overall increase in ASV taxa observed as well as the corresponding respiration and biomass measurements, we estimated the respiration normalized by biomass as a proxy for potential CUE from the incubations. Within each moisture treatment, chitin amended samples had lower CUE than samples without chitin, despite greater resource access. This is because the resource to energy allocation for complex carbon metabolism requires the production of multiple enzymes, reducing the efficiency of generating biomass [55]. Overall, the high moisture samples had increased estimated CUE compared to the low moisture samples. This increased CUE response to increased moisture has been observed in sandy loam soils [56], attributed to the changes in substrate diffusion across the different moisture levels. Here, we demonstrate the importance of soil moisture levels and variable access to resources in modulating enzyme production and CUE.

### Microbial functional redundancy identified through activity-labeling

Although metagenomes, metatranscriptomes, and metaproteomes provide system-level details and clues to the functional roles of community members [57–61], assigning real-time activity to specific taxa at the moment of sampling has been difficult to achieve. To determine microbial members that were directly active in the decomposition of chitin, we extracted soil microbes and applied ABPs targeting membrane-bound or intracellular N-acetyl-β-D-glucosaminidases (NAG-ABP) or endochitinases (Chi3-ABP) for the first time in environmental samples. Secreted, extracellular chitinase would be missed in our ABP-FACS workflow but detected in proteomic-focused experiments [23] on extracellular enzymes or “secretome”, and thus our whole cell labeling workflow is biased toward cell-bound or intracellular enzymes. We also acknowledge the potential underrepresentation of *Actinobacteriota*, which are important for extracellular enzyme production and the degradation of organic compounds, including chitin and chitosan [62]. Our results reveal low level enzyme activity by diverse soil bacteria, reflecting the wide phylogenetic distribution of chitin degrading enzymes [63, 64]. Yet like culturing experiments [13, 65], a small number of soil microbial species generate most of the enzyme activity. By demonstrating the enzymatically functioning members of the soil microbiome community, ABP-labeling has the potential to advance current ecological modeling by focusing in on members actively performing a phenotype *in situ*.

Microbes from three genera were identified as having active cell bound endochitinases (Chi3-ABP labeled) with higher abundance levels in chitin amended samples compared to unamended samples. Among these three genera, *Chitinibacter* had the highest relative abundance followed by *Cellvibrio* and *Massilia*. The abundances for N-acetyl-β-D-glucosaminidase active microbes (NAG-ABP labeled) were also dominated by *Chitinibacter*. Various members of *Chitinibacter* have been isolated from environmental samples using chitin as a substrate [66–69]. This suggests a wide environmental and functional niche for *Chitinibacter* in response to organic matter deposition in soil. *Cellvibrio* was lower in abundance for NAG-ABP than in Chi3-ABP, whereas *Massilia* had slightly higher abundance for NAG-ABP. The differences between NAG-ABP and Chi3-ABP labeled microbes provide evidence of expressed functional redundancy in a complex soil sample. Chitinolytic activity of these genera from soil samples has been previously shown in growth experiments, further supporting the specificity of ABPs for enriching microbes with a chitinolytic phenotype [16, 69]. Wieczorek et al [16] used ^13^C chitin to label bacteria decomposing chitin in a soil slurry collected from an agricultural field and detected both *Cellvibrio* and *Massilia* at time points similar to our seven-day incubation, suggesting primary degradation of chitin by these organisms. A previous study observed a strong response of *Massilia* to chitin amendment, increasing in abundance over 16X from native soil to amended soil [70]. *C. japonicus* has been shown to contain multiple chitinase genes, with detected chitinases with varying levels of activity toward chitin decomposition, suggesting distinct roles of chitinases within one cell population [71].

Due to the complex structure of chitin and other polysaccharides, microbial metabolic heterogeneity of microbial communities has been observed as a viable decomposition strategy [72, 73]. The variation observed among genera for the Chi3- and NAG-ABP, supports the functional redundancy for these genera in a complex soil system. Although certain microbes were characterized with both of the ABP functions, the variability in the relative abundances of cells producing endochitinase or β-D-glucosaminidase enzymes suggests population separation into different metabolic strategies [74]. *Cellvibrio* was identified as having a high abundance using the Chi3-ABP but exhibited a decreased abundance when labeled with NAG-ABP, supporting a dual functionality role with a preference for oligomers of chitin under the experimental conditions, including chitin addition. The substrate specificity of our ABPs allows for the distinction of target molecules for the different steps of organic matter decomposition. High production of N-acetyl-β-D-glucosaminidases and endochitinases by *Chitinibacter* in our chitin amended incubations suggest a broad metabolic lifestyle, expressing the enzymatic machinery for multiple chitin-degrading metabolisms [75, 76].

## Conclusion

Our current understanding of microbial mediated SOM decomposition is largely based on potential enzyme assays in bulk soil and genomic potential from metagenomic surveys. Connecting bulk process rates with taxon-specific enzyme production is important for understanding what drives community responses and phenotypes. In this study, we applied a cell tagging approach to enrich and quantify soil bacterial taxa that are actively producing chitin-degrading enzymes. we showed that historical field soil moisture level and chitin amendment had varying impacts on a multitude of biogeochemical parameters and functions of the microbial community. The interplay of moisture and substrate highlights the complexity of organic matter decomposition and the dichotomous response of microbes to changing conditions. Our application of ABP-FACS in tandem with biogeochemical assays in soil provided quantitative evidence that a subset of microbes produce cell bound chitinolytic enzymes and suggests that most of the measured potential activity in bulk soil is due to extracellular or relic enzymes. Additionally, using ABPs, variations in cell-specific phenotypes were detected across changing soil conditions. Despite widespread functional redundancy, a few key taxa were responsible for the decomposition of chitin with different levels of chitinolytic activity depending on soil moisture levels.

## Supplementary Material

Supplemental_Figure_1_revised_png_wraf250

Supplemental_Figure_2_wraf250

Supplemental_Figure_3_wraf250

Supplemental_Figure_4_revised_png_wraf250

Supplemental_Figure_5_revised_png_wraf250

Supplemental_Figure_6_revised_png_wraf250

manuscript_tables_revised_wraf250

## Data Availability

All sequence data associated with this study have been deposited in Datahub at PNNL (doi.org/10.25584/2475041). Additional raw data files are available through Datahub at PNNL (doi.org/10.11578/2572145). Associated processed data files are available as supplemental online information.
