# Supplementary figures and images for: Impact of moisture on microbial decomposition phenotypes and enzyme dynamics
Source: ISME J. 2025 Nov 15;19(1):wraf250. doi: 10.1093/ismejo/wraf250 (PMC12649754; doi:10.1093/ismejo/wraf250)

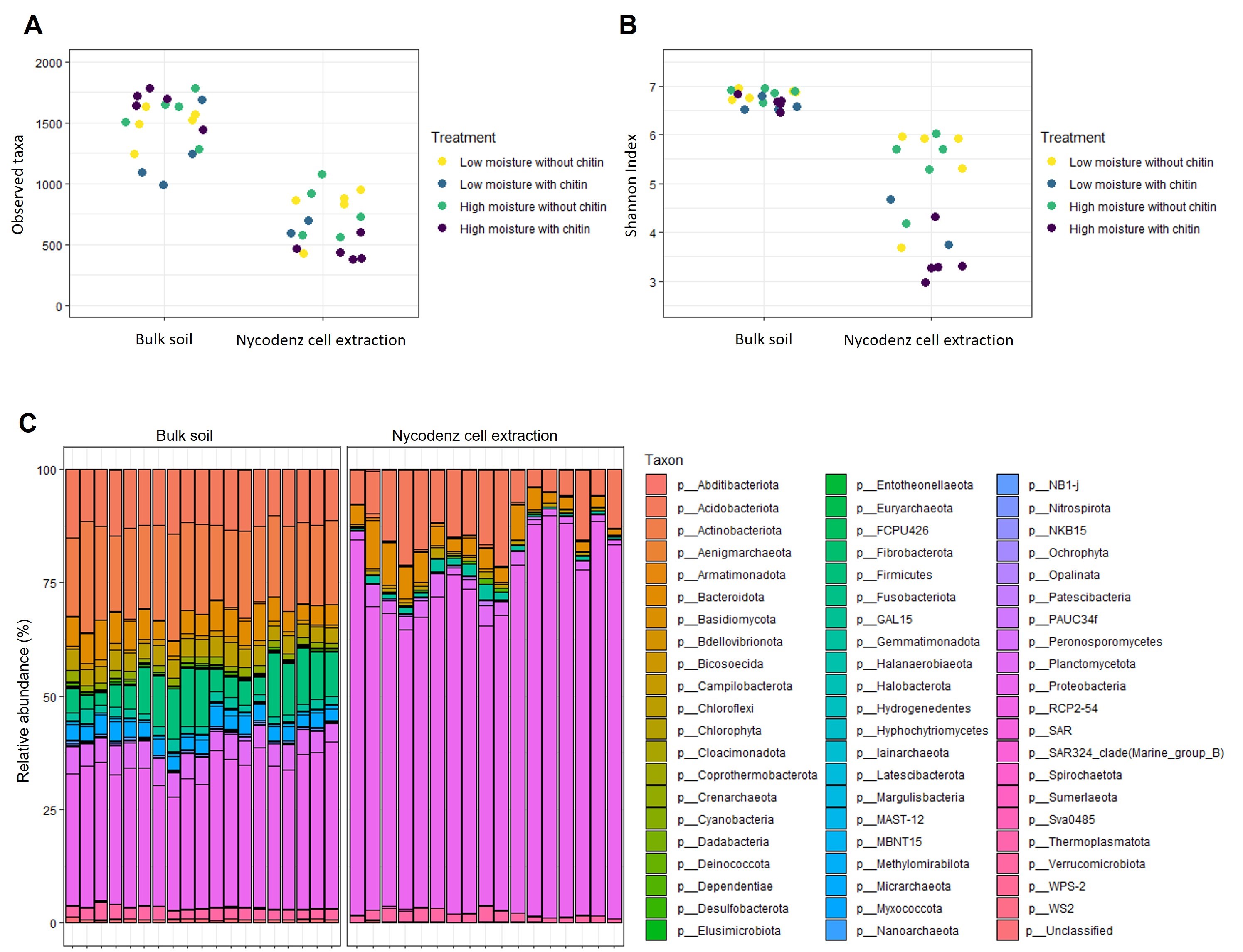

Supplement: Supplemental_Figure_1_revised_png_wraf250 [file supplemental_figure_1_revised_png_wraf250.jpeg]

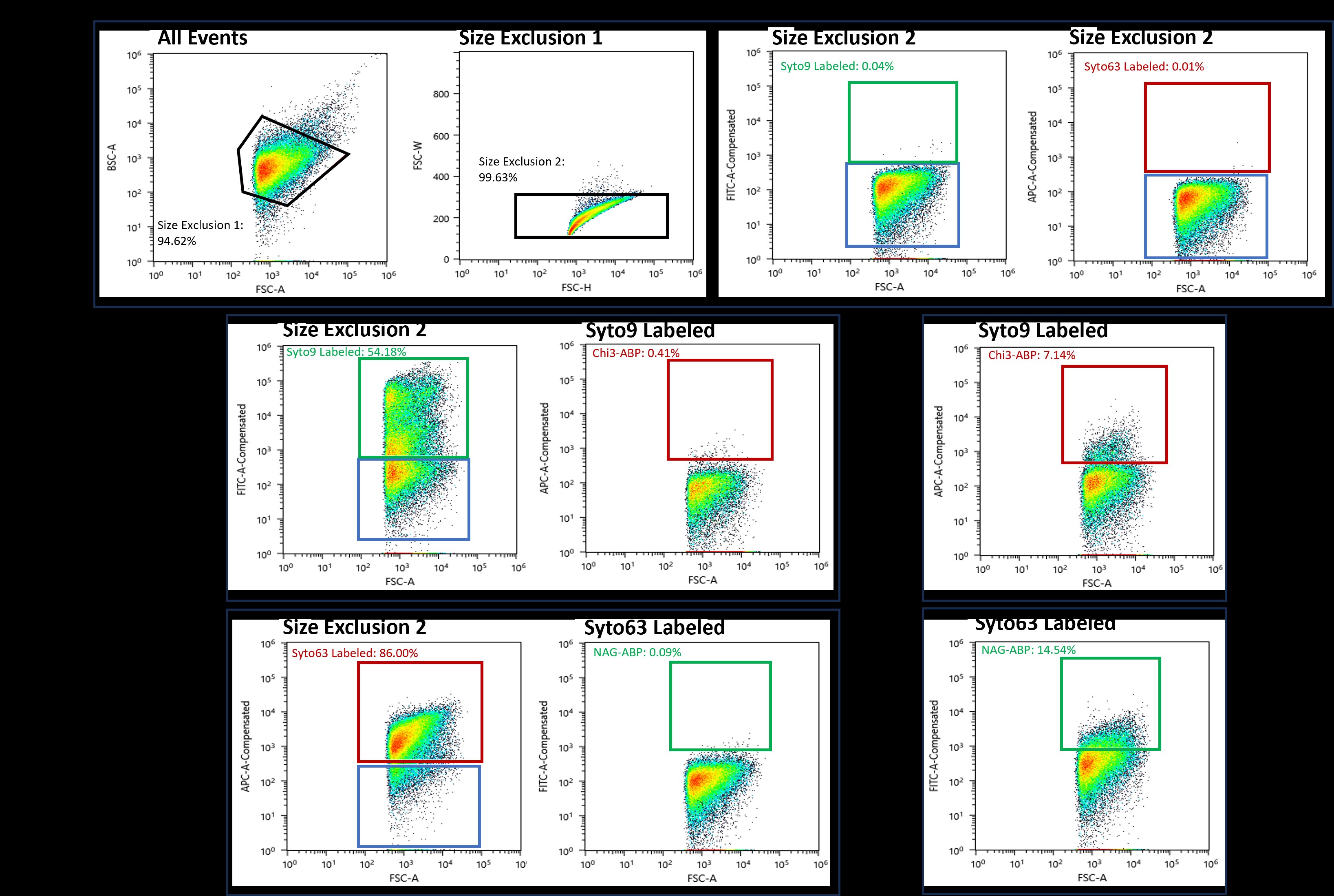

Supplement: Supplemental_Figure_2_wraf250 [file supplemental_figure_2_wraf250.jpeg]

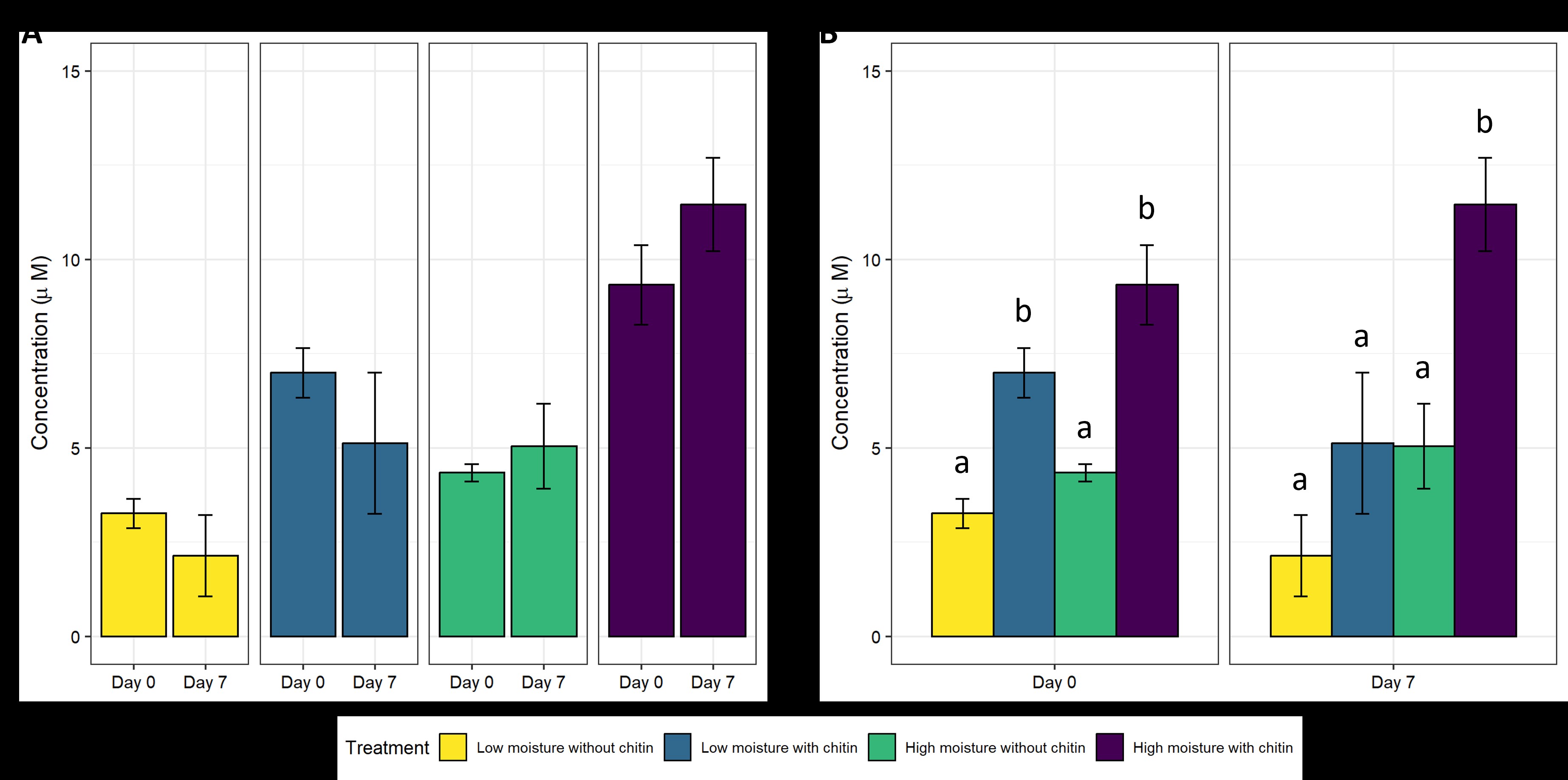

Supplement: Supplemental_Figure_3_wraf250 [file supplemental_figure_3_wraf250.jpeg]

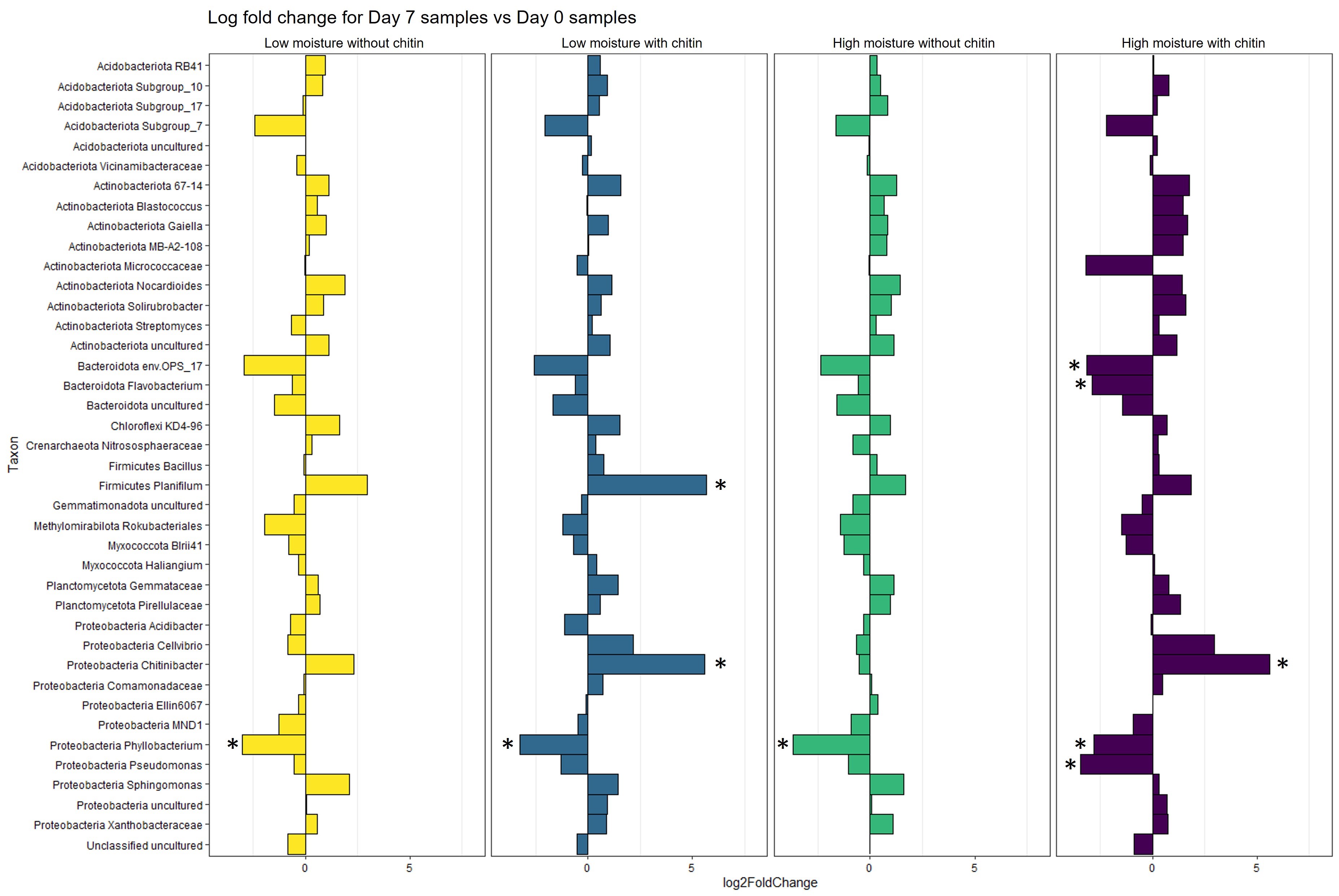

Supplement: Supplemental_Figure_4_revised_png_wraf250 [file supplemental_figure_4_revised_png_wraf250.jpeg]

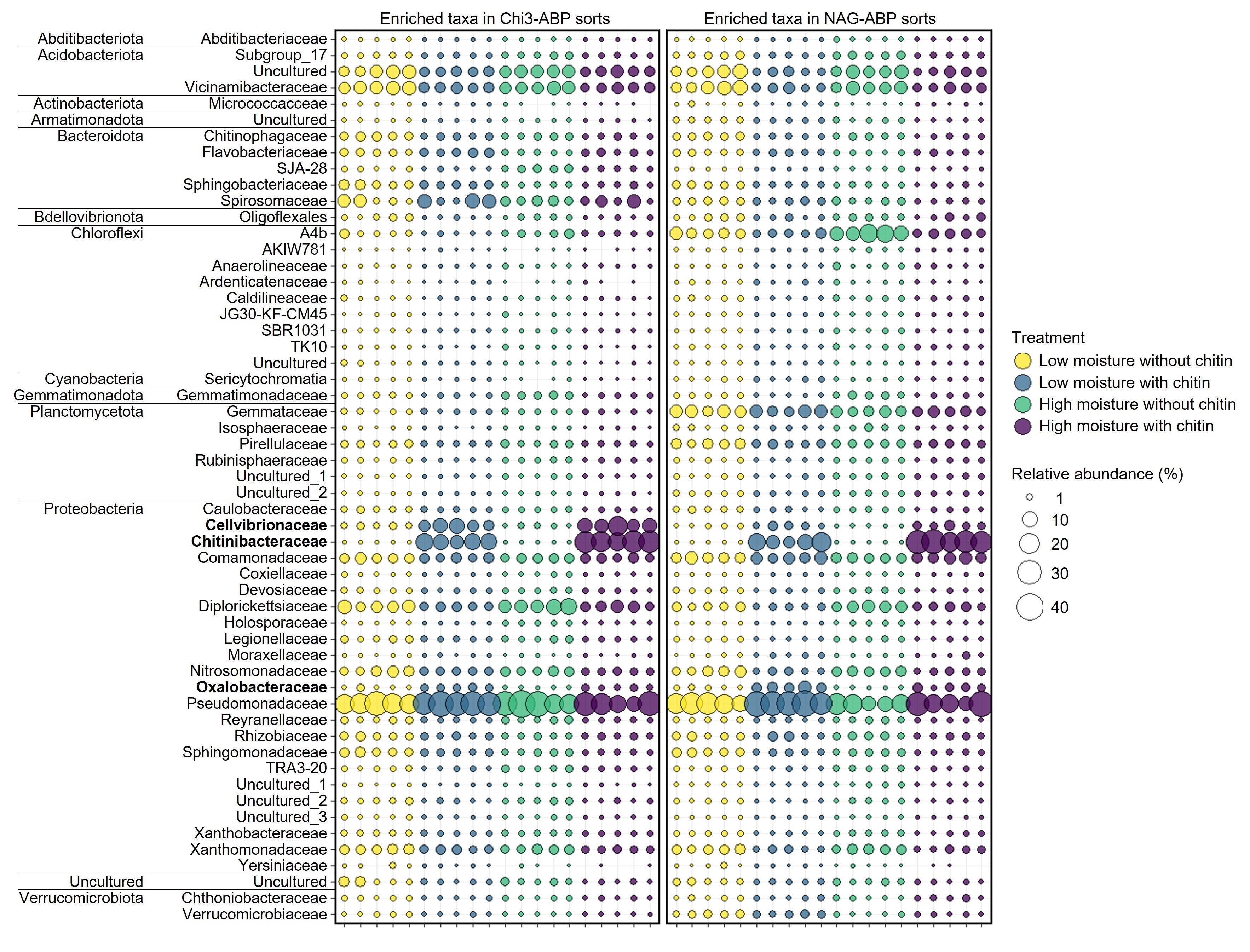

Supplement: Supplemental_Figure_5_revised_png_wraf250 [file supplemental_figure_5_revised_png_wraf250.jpeg]

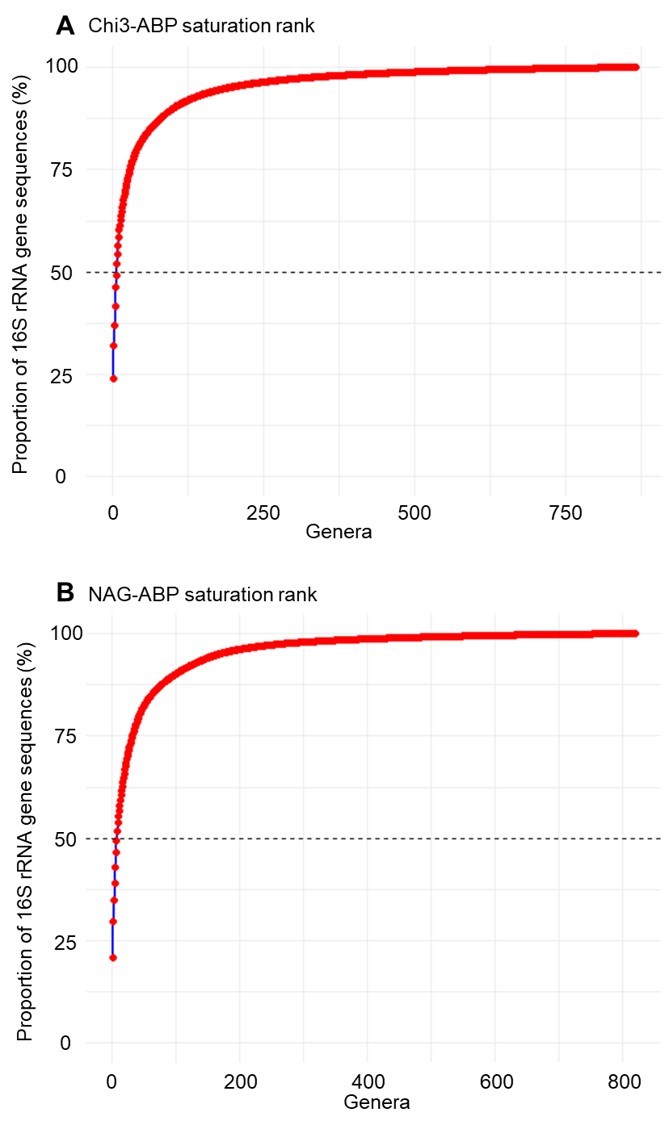

Supplement: Supplemental_Figure_6_revised_png_wraf250 [file supplemental_figure_6_revised_png_wraf250.jpeg]
